# Arborescent Unimolecular Micelles: Poly(γ-Benzyl l-Glutamate) Core Grafted with a Hydrophilic Shell by Copper(I)-Catalyzed Azide–Alkyne Cycloaddition Coupling

**DOI:** 10.3390/polym9100540

**Published:** 2017-10-23

**Authors:** Mario Gauthier, Greg Whitton

**Affiliations:** Department of Chemistry, Institute for Polymer Research and Waterloo Institute for Nanotechnology, University of Waterloo, 200 University Avenue West, Waterloo, ON N2L 3G1, Canada; gwhitton@uwaterloo.ca

**Keywords:** dendritic graft polymers, CuAAC coupling, unimolecular micelles, amphiphilic copolymers

## Abstract

Amphiphilic copolymers were obtained by grafting azide-terminated polyglycidol, poly(ethylene oxide), or poly(2-hydroxyethyl acrylate) chain segments onto alkyne-functionalized arborescent poly(γ-benzyl l-glutamate) (PBG) cores of generations G1–G3 via copper(I)-catalyzed azide–alkyne Huisgen cycloaddition (CuAAC) coupling. The alkyne functional groups on the arborescent PBG substrates were either distributed randomly or located exclusively at the end of the chains added in the last grafting cycle of the core synthesis. The location of these coupling sites influenced the ability of the arborescent copolymers to form unimolecular micelles in aqueous environments: The chain end grafting approach provided enhanced dispersibility in aqueous media and favored the formation of unimolecular micelles in comparison to random grafting. This is attributed to a better defined core-shell morphology for the copolymers with end-grafted shell segments. Aqueous solubility also depended on the type of material used for the shell chains. Coupling by CuAAC opens up possibilities for grafting a broad range of polymers on the arborescent substrates under mild conditions.

## 1. Introduction

**Figure 1:**
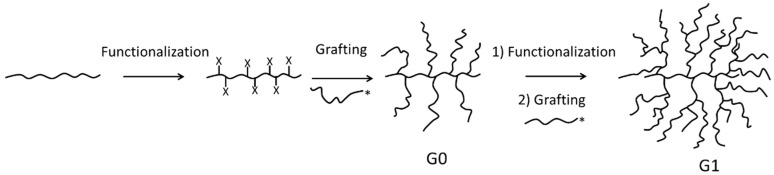
Schematic representation of the generation-based synthesis of arborescent polymers.

Dendrigraft (arborescent) polymers are dendritic molecules synthesized by a strategy similar to dendrimers, but with the notable distinction that polymeric building blocks are used rather than low molecular weight monomers. These polymers, synthesized by Gauthier and Mӧller in 1991 [1], are obtained by a *grafting onto* scheme, whereby well-defined linear side chains are first coupled with a randomly functionalized linear substrate to obtain a comb-branched or generation zero (G0) arborescent polymer. Through additional cycles of substrate functionalization and grafting, the subsequent generations of arborescent polymers are produced as shown in Figure 1. Arborescent polymers with high molecular weights (*M*_n_ > 10^6^ g/mol) and narrow molecular weight distributions (*M*_w_/*M*_n_ ≈ 1.1) are, thus, obtained in only a few reaction cycles [2]. This has been achieved mainly by coupling living anionic polymers with suitably functionalized substrates [1,2,3,4]. While this method takes advantage of the high reactivity of living polymers, allowing fast and efficient coupling, these reactions must be performed under high purity and inert conditions, which limits their scope. The synthesis of arborescent poly(γ-benzyl l-glutamate) (PBG) was achieved more recently by ring-opening polymerization of benzyl l-glutamic acid *N*-carboxyanhydride and carbodiimide peptide coupling techniques [5]. Both the substrates and the side chains can be isolated and stored prior to use, and tolerance to impurities is higher for carbodiimide-mediated coupling than for anionic grafting, albeit this reaction remains sensitive to side reactions. It would thus be beneficial to achieve the synthesis of arborescent polymers by a *grafting onto* scheme using reactive functionalities tolerant to less stringent reaction conditions, and with a wider range of side chain compositions.

The term “click” chemistry was coined in 2001 by Sharpless et al. to describe reactions that are, among others, modular, wide in scope, give very high yields, and generate only inoffensive by-products [6]. These criteria are met by the copper(I)-catalyzed azide–alkyne Huisgen cycloaddition (CuAAC) reaction, developed concurrently by the groups of Meldal [7] and Sharpless [8] in 2002. Meldal et al. used the CuAAC reaction to synthesize peptidotriazoles on solid supports, but were unaware of its link to “click” chemistry. Sharpless et al. more clearly realized the potential of this reaction and described it as having an “unprecedented level of selectivity, reliability, and scope for those organic synthesis endeavors which depend on the creation of covalent links between diverse building blocks” [8]. Since the application of the CuAAC reaction to polymer synthesis by Hawker and coworkers [9] in 2004, various macromolecular architectures have been constructed by this method [10,11]. Due to the applicability of the CuAAC reaction in a broad range of solvents, it is also more tolerant to functional groups and impurities.

The CuAAC reaction was combined with the ring-opening polymerization of α-amino acid *N*-carboxyanhydrides to synthesize amphiphilic block copolymers containing poly(γ-benzyl l-glutamate) (PBG) components. Lecommandoux and coworkers, thus, used CuAAC chemistry to link homopolymers of PBG with poly(2-(dimethylamino)ethyl methacrylate) [12], poly(trifluoroacetyl l-lysine) [13], or dextran [14] and generated well-defined (*M*_w_/*M*_n_ < 1.20) amphiphilic block copolymers that self-assembled into micellar structures. Engler et al. also used CuAAC to generate comb-branched polymers from a linear poly(γ-propargyl l-glutamate) substrate and azide-terminated poly(ethylene oxide) side chains, demonstrating the efficiency of the *grafting onto* approach with grafting yields >95% [15]. The solubility characteristics of the graft copolymers were not investigated however.

It was recently shown that well-defined (*M*_w_/*M*_n_ ≤ 1.22) amphiphilic copolymers could be synthesized by coupling arborescent PBG substrates with linear side chains via carbodiimide-mediated coupling [16]. Reported herein is a new strategy for producing amphiphilic arborescent copolymers by CuAAC grafting. Alkyne-functionalized arborescent PBG substrates were prepared by reacting propargylamine with carboxylic acid functionalities on PBG substrates. These substrates were then coupled with either α-azido polyglycidol (PGly), ω-azido poly(ethylene oxide) (PEO), or ω-azido poly(2-trimethylsilylethyl acrylate) (P(HEA-TMS)) linear chain segments to generate amphiphilic copolymers capable of forming water-soluble unimolecular micelles. A 1:1 ratio of alkyne to azide groups was used in all the reactions, so as to investigate the influence of the side chain composition and the substrate characteristics (generation number and location of the coupling sites) on the grafting efficiency. The focus of the investigation is on the synthetic aspects, but the solution properties of the micelles obtained were also examined using dynamic light scattering (DLS) measurements.

## 2. Materials and Methods

### 2.1. Characterization and Sample Preparation

^1^H Nuclear magnetic resonance (NMR) spectroscopy was performed on a Bruker 300 MHz instrument (Bruker Ltd., Milton, ON, Canada), at a sample concentration of 15–20 mg/mL.

Infrared analysis was performed on a Bruker Vector 22 FT-IR spectrometer (Bruker Ltd., Milton, ON, Canada), supplied with the OPUS 6.0 software package, to acquire and manipulate the spectra. The analysis was performed with 64 scans from 400 to 4000 cm^−1^ at 1 cm^−1^ resolution.

Size exclusion chromatography (SEC) analysis was done on a Viscotek GPCmax instrument (Malvern, Montreal, QC, Canada) with a TDA 305 triple detector array and a Viscotek UV Detector 2600. Three PolyAnalytik Superes™ Series linear mixed bed columns of 300 × 8 mm^2^, with polystyrene molecular weight exclusion limits of 400 × 10^3^, 4 × 10^6^, and 20 × 10^6^ g/mol (PolyAnalytik, London, ON, Canada) were used in series with tetrahydrofuran (THF) as the mobile phase at a flow rate of 1.0 mL/min and 35 °C. Analysis of the PEO and P(HEA-TMS) linear chains, and all the arborescent copolymers were performed on a SEC instrument with a Waters 510 HPLC pump (Waters Ltd., Mississauga, ON, Canada), a 50 μL injection loop, a Waters 2410 differential refractometer (DRI) detector (Waters, Mississauga, ON, Canada), and a Wyatt MiniDAWN laser light scattering detector (Wyatt Technology Corp., Santa Barbara, CA, USA) operating at 690 nm to determine the absolute molecular weight of the polymers. The column was a 500 × 10 mm^2^ Jordi Gel Xstream H2O Mixed Bed model (Jordi Labs, Mansfield, MA, USA) with a polystyrene molecular weight range of 10^2^–10^7^ g/mol. *N,N*-Dimethylformamide (DMF) with LiCl (1 g/L, to minimize polymer adsorption) was used at 1.0 mL/min and room temperature (RT). Knowledge of the refractive index increment (d*n*/d*c*) of linear α-azido polyglycidol, ω-azido poly(ethylene oxide) and ω-azido poly(2-trimethylsilylethyl acrylate) was necessary to determine the absolute molecular weight by SEC. These were determined on a Brookhaven Instruments BI-DNDC 620 differential refractometer (Brookhaven Instruments Corp., Holtsville, NY, USA), using five solutions in DMF at concentrations ranging from 1 to 5 mg/mL at 30 °C. Preparative SEC was carried out with a Waters M45 pump (Waters Ltd., Mississauga, ON, Canada), a 2-mL sample injection loop, a Waters R401 differential refractometer detector (Waters Ltd., Mississauga, ON, Canada), and a Jordi Gel DVB 1000 Å (Jordi Labs, Mansfield, MA, USA) or a Mixed Bed 250 × 22 mm^2^ column (Jordi Labs, Mansfield, MA, USA). DMF with 0.2 g/L LiCl at 3.0 mL/min and RT served as the mobile phase, and the polymer was injected as a 20–30 mg/mL solution.

Dynamic light scattering measurements were carried out batch-wise on a Brookhaven BI-200SM light scattering goniometer (Brookhaven Instruments Corp., Holtsville, NY, USA) with a BI-APD (avalanche photo diode) detector and a Claire Lasers CLAS2-660-140C (120 mW) laser (Claire Lasers Corp., Kitchener, ON, Canada) operating at 660 nm. All the samples were measured at 25 °C and a scattering angle of 90° after filtration twice with 3.0 μm polytetrafluoroethylene (PTFE) membrane filters. The correlator was operated in the exponential sampling mode and the hydrodynamic diameters were calculated from the *Z*-average translational diffusion coefficients obtained from first- and second-order cumulant analysis of the correlation function, to better account for polydispersity effects. CONTIN analysis of the data was also used to generate size distributions for the samples. Solutions were prepared at 0.1–2% *w*/*v*, depending on the molecular weight (generation number) of the sample. If solvent exchange was necessary, 3 mL of a sample solution was placed in a 12,000–14,000 g/mol molecular weight cut-off (MWCO) regenerated cellulose dialysis bag overnight in at least 200 mL of the new solvent. The solvent was replaced on the next day and stirring was continued for at least 2 h longer to ensure complete removal of the original solvent.

### 2.2. Solvent and Reagent Purification

*N*,*N*-Dimethylformamide (DMF; peptide synthesis grade, Sigma-Aldrich, Oakville, ON, Canada) was distilled under reduced pressure and stored in the dark. *n*-Hexylamine (99%, Sigma-Aldrich, Oakville, ON, Canada) was stirred overnight with CaH_2_ and distilled under reduced pressure. The purified DMF and *n*-hexylamine were stored under nitrogen (N_2_) over 3 Å molecular sieves (EMD Millipore, Etobicoke, ON, Canada). The toluene (ACS Reagent, Sigma-Aldrich, Oakville, ON, Canada) used for the anionic polymerization of glycidol acetal was distilled over oligostyryllithium under N_2_. All the other reagents and solvents were used as received from the suppliers.

### 2.3. Synthesis of Linear Polymers with a Terminal Azide Functionality

#### 2.3.1. Synthesis of α-Azido PGly

The monomer 2,3-epoxy-1-(1-ethoxyethoxy)propane (glycidol acetal) was synthesized as described by Fitton et al. [17] and used to prepare α-azido-poly(glycidol acetal) with a target *M*_n_ = 10,000 g/mol (PGlyAc) by a procedure adapted from Gervais et al. [18]. Removal of the acetal protecting groups by a procedure adapted from Mendrek et al. [19] yielded the corresponding deprotected polyglycidol, α-azido-PGly. Detailed procedures for the synthesis of the polymers are provided in the Supporting Information.

#### 2.3.2. Synthesis of ω-Azido PEO

Linear PEO monomethyl ether with *M*_n_ = 5000 g/mol (Polysciences, Warrington, PA, USA; *X*_n_ = 113) was subjected to tosylation twice with *p*-toluenesulfonyl chloride to achieve full conversion, and the tosyl group was then displaced with sodium azide as described in the Supporting Information to obtain the azide-terminated polymer.

#### 2.3.3. Synthesis of ω-Azido P(HEA-TMS)

To achieve the controlled polymerization of 2-hydroxyethyl acrylate (HEA) via atom transfer radical polymerization (ATRP), the hydroxyl group was protected as a trimethylsilyl ether to yield 2-(trimethylsilyloxy)ethyl acrylate (HEA-TMS) by a procedure adapted from Mühlebach et al. [20]. The ATRP of HEA-TMS to obtain ω-bromo P(HEA-TMS) with a target *X*_n_ = 50 was conducted using copper(I) bromide, *N*,*N*,*N*′,*N″*,*N″*-pentamethyldiethylenetriamine (PMDETA) and methyl 2-bromopropionate. The ω-bromo P(HEA-TMS) thus obtained was converted to the ω-azido derivative by displacement with sodium azide. Details on these reactions can be found in the Supporting Information.

### 2.4. Synthesis of CuAAC-Grafted Arborescent Copolymers

#### 2.4.1. Synthesis of Alkyne-Functionalized Arborescent PBG Cores

The synthesis of the arborescent PBG substrates, either randomly or chain end-functionalized with carboxylic acid groups, was described earlier [16]. To obtain alkyne-functionalized arborescent PBG substrates, propargylamine was coupled with the carboxylic acid moieties using *N*,*N*′-diisopropylcarbodiimide (DIC) and 1-hydroxybenzotriazole (HOBt). For example, chain end carboxylic acid-functionalized G1PBG (0.202 g, 6.64 × 10^−5^ mol CO_2_H, 1 eq.) was placed in a 10-mL round bottomed flask with 4 mL of dry DMF. DIC (52 μL, 3.32 × 10^−4^ mol, 5 eq.) and HOBt (45 mg, 3.32 × 10^−4^ mol, 5 eq.) were then added to the reaction, followed by propargylamine (8 μL, 1.33 × 10^−4^ mol, 2 eq., 98%, Sigma-Aldrich, Etobicoke, ON, Canada). The reaction was stirred overnight under N_2_ at RT. The crude product was purified by preparative SEC in DMF, to ensure the complete removal of excess propargylamine. The purified product was concentrated to 4–6 mL, precipitated in methanol, centrifuged, and dried overnight under vacuum. Yield: 0.153 g (76%). SEC (DMF): *M*_n_ = 280,000 g/mol, *M*_w_/*M*_n_ = 1.05 (MALLS). ^1^H NMR (300 MHz, *d*_6_-DMSO) δ: 8.2–7.8 (b, 1H), 7.28–7.20 (s, 5H), 5.03–4.89 (s, 2H), 4.35–3.80 (b, 1H), 3.80–3.70 (b, 2H), 3.10–2.90 (b, 1H), 2.33–1.70 (b, 4H), 1.35–1.10 (b, 10H), 0.80–0.70 (b, 3H).

#### 2.4.2. Synthesis of CuAAC-Grafted Arborescent Copolymers

As an example, chain end alkyne-functionalized G1PBG (30.1 mg, 9.78 × 10^−6^ mol alkyne, 1 eq.), α-azido PGly (69 mg, 9.78 × 10^‒6^ mol N_3_, 1 eq.), and CuSO_4_ (4.9 mg, 1.96 × 10^−5^ mol, 2 eq.) were dissolved in 3 mL of DMF in a dry Schlenk flask with a magnetic stirring bar. One freeze-pump-thaw (FPT) cycle was performed and sodium l-ascorbate (NaAsc, 7.7 mg, 3.91 × 10^−5^ mol, 4 eq., ≥98%, Sigma-Aldrich, Etobicoke, ON, Canada) was added under N_2_. One more FPT cycle was performed and the flask was purged with N_2_ before stirring at RT. After 24 h the solution was diluted with DMF containing 0.2 g/L LiCl and the copolymer was purified by preparative SEC. SEC (DMF): Grafting yield = 60% (DRI), *M*_n_ = 906,000 g/mol, *M*_w_/*M*_n_ = 1.09 (MALLS).

## 3. Results and Discussion

### 3.1. Synthesis of Linear Polymers with a Terminal Azide Functionality

Three types of linear polymers were synthesized to couple with the hydrophobic arborescent PBG cores and generate a hydrophilic shell. The characterization data obtained for the linear polymers are provided in Table 1. All the azide-terminated polymers had narrow molecular weight distributions, indicative of well-controlled polymerization and/or modification reactions. A detailed discussion of the synthesis of these polymers is provided in the Supporting Information.

### 3.2. Synthesis of Alkyne-Functionalized Arborescent PBG Cores

**Figure 2:**
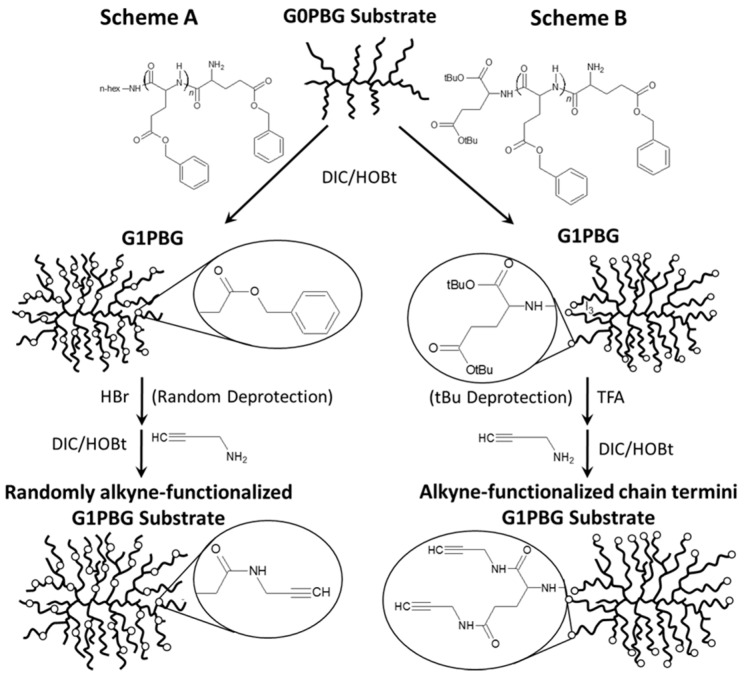
Schematic representation of the synthesis of (**A**) randomly alkyne-functionalized and (**B**) chain termini alkyne-functionalized G1PBG substrates starting from a partially-deprotected (randomly carboxylic acid-functionalized) G0PBG substrate. The synthesis of the G1PBG intermediates involves coupling the G0PBG substrate with either non-functional (A) or di(*t*-butyl ester)-functionalized (B) linear PBG chains.

Alkyne-functionalized PBG substrates were obtained by reacting the carboxylic acid groups on the substrates with propargylamine via carbodiimide activation. The synthesis of arborescent G1PBG, either randomly or chain end-functionalized with carboxylic acids, was discussed in detail in an earlier publication [16]. A schematic representation of the preparation of the two types of alkyne-functionalized G1PBG substrates is provided in Figure 2 as an example. Both syntheses start from a G0PBG substrate randomly functionalized with carboxylic acid groups, obtained by coupling linear partially-deprotected PBG with linear PBG side chains, followed by partial deprotection [16]. In Figure 2A, a G1PBG substrate with randomly-distributed carboxylic acid groups is generated by grafting non-functional (hexyl-terminated) linear PBG on the G0 substrate and partial deprotection with HBr. In Figure 2B, PBG side chains derived from an initiator with two *tert*-butyl ester-protected carboxylic acid functionalities are used to obtain the G1PBG substrate. This allows selective deprotection of the *tert*-butyl ester protecting groups at the chain ends with trifluoroacetic acid (TFA). A one-fold excess of propargylamine was added in the reaction to ensure complete conversion of the carboxylic acid groups to alkyne amide functionalities. The carboxylic acid functionalities on the substrates were easily accessible to the small molecule propargylamine, so that complete conversion was expected. For convenience, a preparative SEC instrument was used to isolate the alkyne-functionalized PBG substrates from excess propargylamine prior to ^1^H NMR analysis, albeit this could also have been achieved by successive precipitations of the sample.

**Figure 3:**
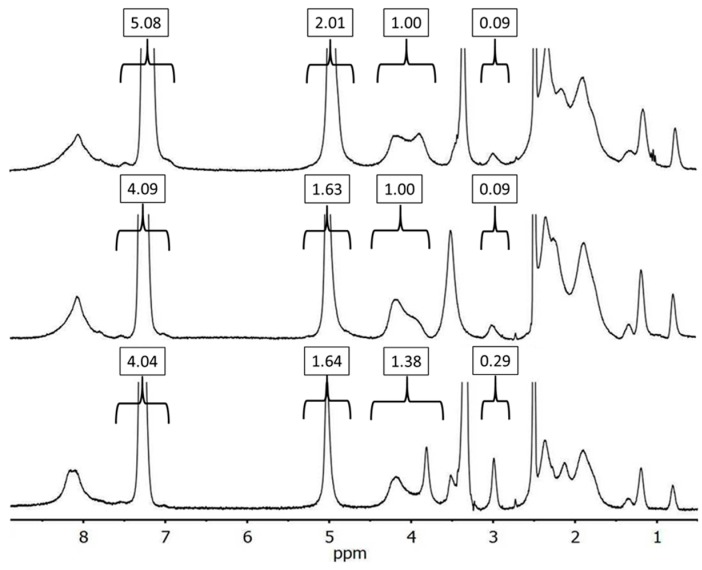
^1^H NMR spectra for (top to bottom) G1PBG substrate, randomly deprotected (19 mol % CO_2_H), and randomly alkyne-functionalized (19 mol % alkyne) in *d*_6_-DMSO.

^1^H NMR spectra illustrating the synthesis of a randomly alkyne-functionalized G1PBG substrate are provided in Figure 3. The spectrum for G1PBG before removal of a portion of the benzyl ester protecting groups is displayed at the top, whereas the spectrum for partially-deprotected G1PBG is shown in the middle. The integrals for the benzyl ester protons at 5.0 and 7.2 ppm decreased in intensity, relatively to the methine proton at 4.4–3.7 ppm (integral value set to 1.00), from 5.08 and 2.01 before deprotection to 4.09 and 1.63 after partial deprotection, respectively. This corresponds to a deprotection level of 19%. The spectrum for randomly alkyne-functionalized G1PBG, provided at the bottom of the figure, has an alkyne proton resonance appearing at 3.0 ppm and a signal for the protons adjacent to the alkyne group near 3.8 ppm. The alkyne substitution level was determined by integration of the signal at 3.0 ppm and the peptidic methine proton peak from 4.4 to 3.7 ppm. An overlapping peak at 3.0 ppm is from *n*-hexylamine, the initiator serving in the ring-opening polymerization of benzyl glutamate to synthesize the PBG substrates. This signal was present before the propargylamine reaction and was, therefore, subtracted from the integration value. The overlapping peaks at 3.8 ppm from the propargylamine protons were likewise subtracted from the peak integral for the methine proton at 4.4–3.7 ppm. The carboxylic acid and alkyne functionality levels determined by that method were both 19 mol %, indicating 100% conversion. 

**Figure 4:**
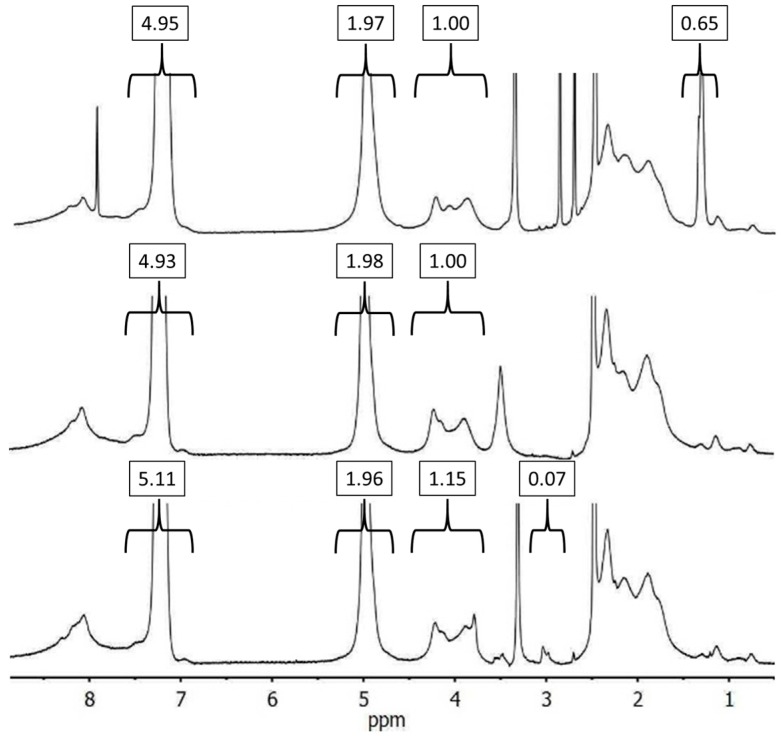
^1^H NMR spectra for (top to bottom) G1PBG with *tert*-butyl-protected chain ends, deprotected chain ends (7 mol % CO_2_H), and alkyne-functionalized chain ends (7 mol % alkyne) in *d*_6_-DMSO.

The ^1^H NMR spectra referring to the synthesis of G1PBG functionalized with alkyne groups at the chain ends are provided in Figure 4. The top spectrum is for the G1 polymer with *tert*-butyl group-protected chain ends, to be selectively deprotected by dissolution in neat TFA (providing carboxylic acid functionalities) while leaving the benzyl ester substituents on the chains unaffected (middle spectrum on Figure 4). The spectrum at the bottom is for G1PBG modified with alkyne chain termini. The same alkyne proton and α-protons appear as in Figure 3, albeit at a lower intensity. An alkyne functionality level of 7 mol % was determined for the chain end-functionalized G1PBG, which also corresponds to 100% conversion.

### 3.3. Optimization of CuAAC Reactions with PBG and Synthesis of G0 Copolymers

All the grafting reactions reported herein used a 1:1 ratio of azide to alkyne functionalities. This ratio was purposely maintained constant in all the reactions, to determine how the efficiency of the coupling reaction was affected by parameters such as the composition of the side chains being grafted, but particularly by the characteristics (generation number and location of the coupling sites) of the arborescent substrates used in the reaction. The yield of the grafting reactions is correspondingly low in some cases, but it is clear that it could be increased by modifying the reaction conditions used (e.g., using a larger ratio of PBG substrate to side chains). In recognition of the lower grafting yields achieved under the conditions selected for this investigation, the grafting procedures are identified as CuAAC coupling reactions rather than “click” reactions, for which consistently higher yields would be expected. The grafting yield (fraction of side chains grafted onto the substrate) was determined from the weight fraction of each component in the copolymers, along with the known amounts of substrate and side chains used in the grafting reaction as described earlier [16]. The solubility characteristics of the PBG substrates limited the solvents for the CuAAC reaction to either DMF or DMSO, and also influenced the selection of the catalyst. Due to its solubility in DMF a combination of CuBr and PMDETA was explored initially, as this catalyst system has been widely used for polymer-related CuAAC reactions [21]. Given the poor results obtained with CuBr/PMDETA, another less reactive catalyst, bromotris(triphenylphosphine) copper(I) bromide, was also investigated but gave even lower grafting yields. It was determined that CuSO_4_ with sodium ascorbate (NaAsc) provided the best results, so these reactions will be discussed in more detail.

The CuSO_4_/NaAsc combination also has been widely used as a source of Cu(I) [22]. This catalyst system is generally employed in aqueous or aqueous/alcoholic mixtures, due to the limited solubility of NaAsc in organic solvents. One benefit of this approach is that the reactions can proceed in the presence of residual oxygen, since NaAsc acts as a reducing agent for copper(II) and can regenerate copper(I) continuously during the reaction. Because NaAsc has limited solubility in DMF, two FPT cycles were, nevertheless, used to remove dissolved oxygen prior to the addition of NaAsc to ensure the presence of a maximum amount of copper(I) catalyst in the reaction. The color of the solution started as greenish blue but turned bright yellow minutes after adding NaAsc, and persisted over the 24 h reaction period. Removal of the copper catalyst by preparative SEC was efficient, as indicated by the absence of color in the purified copolymer solutions. This synthetic protocol was, therefore, applied to the preparation of the G0PBG copolymers with the different α-azido side chains.

**Figure 5:**
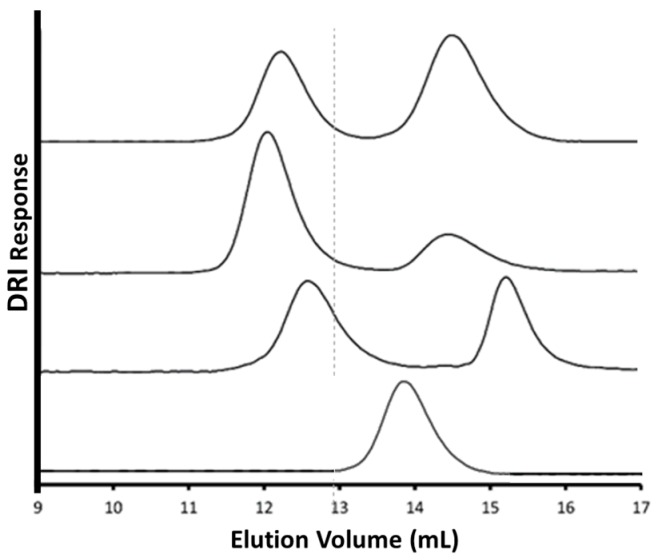
SEC traces in DMF with 0.1% LiCl for (top to bottom) G0PBG-*g*-P(HEA-TMS), G0PBG-*g*-PGly, G0PBG-*g*-PEO after 24 h using CuSO_4_/NaAsc, and G0PBG.

The characteristics of the purified arborescent copolymers derived from randomly alkyne-functionalized G0PBG substrates coupled with ω-azido P(HEA-TMS), α-azido PGly, and ω-azido PEO are summarized in Table 2, and the SEC traces obtained for the crude copolymers and the G0 substrate are compared in Figure 5. The peak for the copolymer (leftmost peak) is shifted relatively to the G0 substrate in all cases, while unreacted P(HEA-TMS), PGly or PEO side chains give the rightmost peak. The SEC peaks obtained for all three arborescent copolymers after 24 h of reaction are relatively symmetrical, without signs of degradation, demonstrating that CuSO_4_/NaAsc was useful under these conditions. The range of grafting yields listed in Table 2 (54–93%) suggests that the nature of the side chains influences the grafting yield. Given the limited amount of data available, it is unclear whether this variation is related to characteristics, such as the chemical composition or the bulkiness of the side chains. While the G0PBG substrate was useful to optimize the CuAAC reaction for the synthesis of arborescent copolymers, coupling of the higher generation substrates of arborescent PBG (G1–G3) with CuSO_4_/NaAsc may provide further insight into these reactions.

### 3.4. Randomly CuAAC-Grafted Arborescent Copolymers from G1 and G2 Substrates

**Figure 6:**
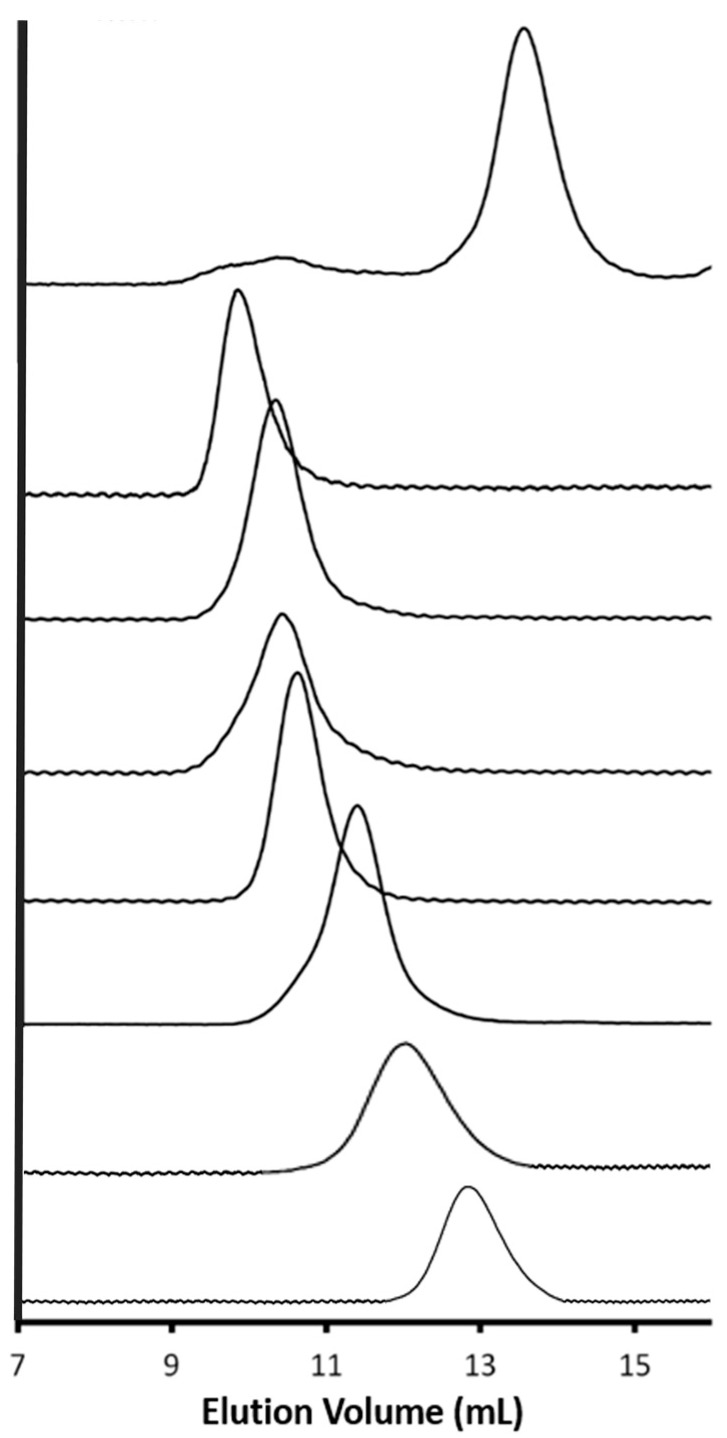
SEC traces in DMF with 0.1% LiCl for (top to bottom) G2PBG-*g*-P(HEA-TMS) crude, G2PBG-*g*-PGly, G2PBG-*g*-PEO, G1PBG-*g*-P(HEA-TMS), G1PBG-*g*-PGly, G1PBG-*g*-PEO, G2PBG, and G1PBG.

The linear side chains with terminal azide functionalities were also grafted onto arborescent G1PBG and G2PBG substrates randomly functionalized with alkyne groups using CuSO_4_/NaAsc. The characteristics of the randomly CuAAC-grafted arborescent copolymers derived from the G1 and G2 substrates are summarized in Table 3. The grafting yield varied between 8–27% in most cases, except for G1PBG-*g*-PEO which had a yield of 57%, while the synthesis of G2PBG-*g*-P(HEA-TMS) was unsuccessful. These relatively low grafting yields were expected due to steric hindrance arising from the compact structure of the arborescent PBG substrates, as previously observed for random grafting reactions via carbodiimide activation [16]. The higher grafting yield observed for G1PBG-*g*-PEO may be linked to the more open structure of G1PBG, together with the high flexibility of the PEO chains. A similar effect was observed previously for carbodiimide-promoted coupling, in that the only successful grafting reaction with a randomly-functionalized G3PBG substrate was for the PEO side chains, with a grafting yield of 58% [16]. The molecular weight distribution of all the arborescent copolymers obtained was relatively narrow (*M*_w_/*M*_n_ ≤ 1.14). The SEC traces for the purified copolymers with P(HEA-TMS), PGly and PEO side chains, the G1PBG and G2PBG substrates are compared in Figure 6, except for the G2PBG-*g*-P(HEA-TMS) sample where only the crude product was recovered since the reaction failed. Relatively symmetrical peaks are observed for all the purified arborescent copolymers, indicating that no degradation occurred during CuAAC grafting.

#### Dynamic Light Scattering Measurements

DLS measurements were performed on the arborescent copolymers in DMF and in aqueous phosphate-buffered saline (PBS) to characterize their solution properties. To obtain a water-soluble PHEA shell, removal of the labile TMS protecting group was achieved by simply adding a few drops of a dilute HCl solution into the DMF solution containing the G1PBG-*g*-P(HEA-TMS) sample and stirring for a few minutes. Both the PGly and PEO shell components are water-soluble and required no further modification. First- and second-order analysis of the correlation function, │g_1_(τ)│ and │g_2_(τ)│, respectively, provide information on the size dispersity of the system. Monodispersed samples are expected to yield identical results for their first- and second-order analysis, since the correlation function can be represented by a single exponential decay under these conditions [23]. Therefore, as the size distribution of a sample broadens or becomes bimodal, the difference between the first- and second-order analysis results increases. The first- and second-order hydrodynamic diameters (*d*_h1_ and *d*_h2_, respectively) obtained for the copolymers and their respective PBG cores in DMF are compared in Table 4. The uncertainties reported are either the standard deviation for a series of 5 measurements or 1 nm, whichever was larger. This approach was used in an earlier investigation [16], together with more detailed CONTIN analysis of the DLS data of obtain size distributions, and it was shown that both methods led to similar conclusions. For the sake of completeness, CONTIN analysis results were also included as Supporting Information (Figures S1 and S2) in the current investigation, and reference is made to these results in support of the conclusions from the simpler analysis based on *d*_h1_ and *d*_h2_ as required. The variability of CONTIN analysis, due to its sensitivity to baseline noise [24] and difficulties encountered in resolving multimodal distributions [25], should be kept in mind when attempting to compare size distributions derived from DLS. Consequently, such comparisons should be considered semi-quantitative at best.

The relatively narrow molecular weight distributions observed in the SEC measurements (*M*_w_/*M*_n_ ≤ 1.14) suggest that the first- and second-order hydrodynamic diameters of the arborescent copolymers should be in close agreement. This is indeed the case for most samples characterized in DMF, since this solvent is relatively good for both the core and the shell components of the molecules. One notable exception is the *d*_h1_ and *d*_h2_ values for G1PBG-*g*-PHEA in DMF, which are much larger than for the G1PBG core in DMF, suggesting that aggregated species were present in that sample even in DMF. The *d*_h1_ and *d*_h2_ values also vary by 14 nm, which is likewise consistent with aggregation. A similar trend is observed for G1PBG-*g*-PGly, with significantly larger *d*_h1_ and *d*_h2_ values and a 13 nm difference. The size distributions for both randomly-grafted copolymers, generated by CONTIN analysis of the DLS data and provided as Supporting Information (Figures S1 and S2), are consistent with these results. Sample G1PBG-*g*-PHEA has the broadest size distribution among all the samples, with sizes spanning a range of over one order of magnitude and an average diameter ca. five-fold larger than the G1PBG substrate (Figure S1). While the size distribution for G1PBG-*g*-PGly is narrower, the distribution is centered at diameters ca. 10-fold larger than the substrate. Aggregation is not expected for arborescent PBG in DMF, but once it is partly deprotected (through loss of the benzyl ester group) its solubility in DMF may be decreased. The aggregation of the G1PBG copolymers in DMF is, therefore, attributed to the fact that they have a rather open structure relatively to the G2PBG copolymers, and the presence of free carboxyl groups, both of which favor the aggregation of the PBG cores in DMF. Indeed, the more highly branched G2PBG-*g*-PGly and G2PBG-*g*-PEO copolymers had *d*_h1_ and *d*_h2_ values in much closer agreement in DMF, and their size distributions were generally narrower and more comparable in size to the substrates (Figure S2). Even for the few randomly grafted copolymer samples that were dispersible in aqueous PBS solutions (G1PBG-*g*-PHEA, G1PBG-*g*-PGly, G2PBG-*g*-PGly), the larger and more variable hydrodynamic diameters and/or bimodal size distributions observed (Figures S1 and S2) were expected based on previous results obtained for copolymers with randomly-grafted shell components [16]. While G1PBG-*g*-PHEA and G1PBG-*g*-PGly both yielded narrow size distributions in aqueous (PBS) environments (Figure S1), the very large diameters observed (upwards of 200 nm) are consistent with large scale aggregation rather than unimolecular micelle formation. The insolubility of the G1PBG-*g*-PEO and G2PBG-*g*-PEO samples was likewise expected based on earlier results [16]. The aggregation and/or poor solubility observed for randomly grafted copolymers in PBS solutions was previously attributed to insufficient shielding of the hydrophobic PBG cores from the aqueous environment, due to a poorly defined (diffuse) core-shell morphology obtained when the hydrophilic shell components are grafted randomly onto the PBG substrates [16].

### 3.5. Chain End CuAAC-Grafted Arborescent Copolymers

Chain end-grafted copolymers derived from arborescent PBG substrates were previously synthesized using peptide coupling techniques [16]. It was shown that chain end grafting provided improved yields and solution properties relatively to random grafting. A similar trend was, therefore, expected for the CuAAC approach. 

**Figure 7:**
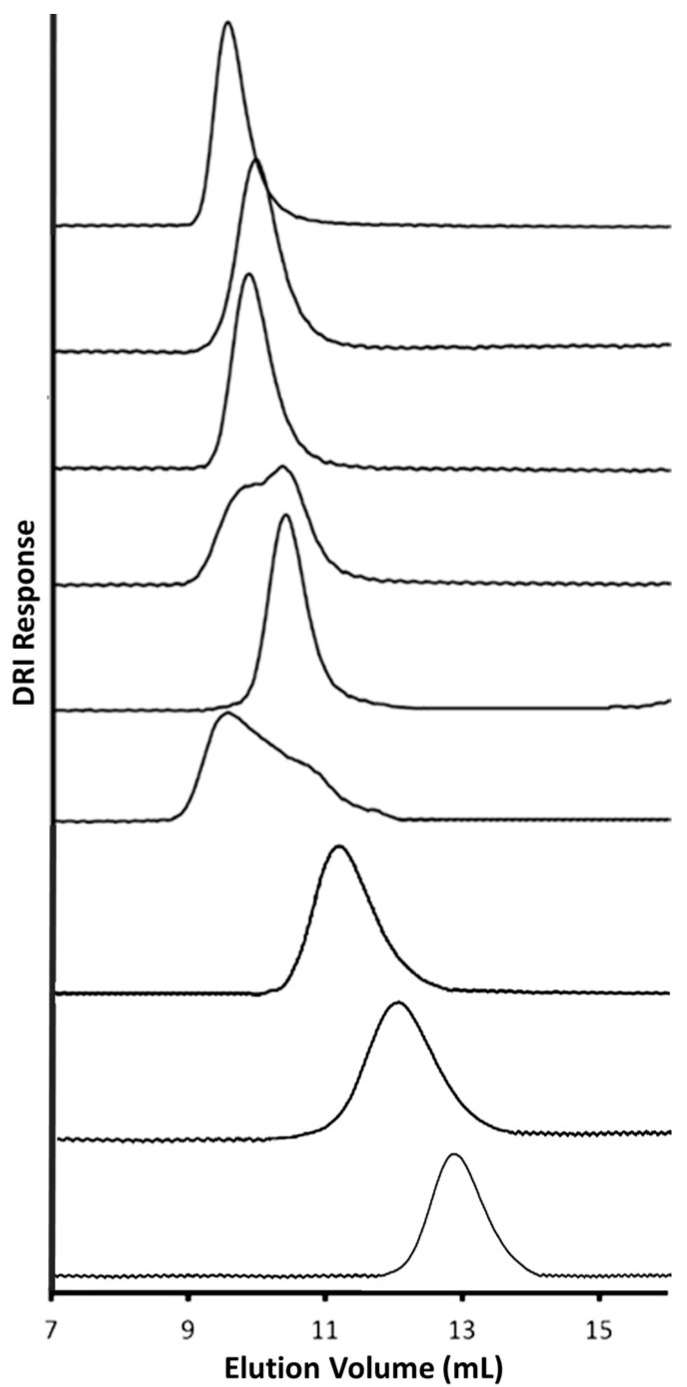
SEC traces in DMF with 0.1% LiCl for (top to bottom) G3PBG-*eg*-PGly, G3PBG-*eg*-PEO, G2PBG-*eg*-PGly, G2PBG-*eg*-PEO, G1PBG-*eg*-PGly, G1PBG-*eg*-PEO, G3PBG, G2PBG, and G1PBG.

CuAAC grafting reactions of P(HEA-TMS) with both the G1PBG and G2PBG chain end-functionalized substrates were attempted but failed, as less than 3% grafting was observed in both cases. The reactions worked for the α-azido PGly and ω-azido PEO chains, however, and the characteristics of the copolymers obtained are summarized in Table 5. The grafting yields observed for the arborescent copolymers using PGly follow the expected trend, with yields decreasing as the substrate generation number increases. The copolymers with PEO had lower grafting yields than those containing PGly. The G1PBG-*eg*-PEO and G2PBG-*eg*-PEO samples also had significantly larger polydispersities of 1.30 and 1.23, respectively, which may lead to inaccurate absolute molecular weight determinations by the SEC-MALLS detector. The SEC traces obtained for the arborescent copolymers and the PBG substrates are compared in Figure 7. Repeat SEC measurements and syntheses for both G1PBG-*eg*-PEO and G2PBG-*eg*-PEO yielded results similar to those in Table 5. It is obvious that the elution volumes for G1PBG-*eg*-PEO and G2PBG-*eg*-PEO are lower than expected, suggesting that either cross-linking has taken place, aggregation or some other artefact is present in the SEC measurements. Since no reliable molecular weight values could be obtained by SEC analysis of the G1PBG-*eg*-PEO and G2PBG-*eg*-PEO samples, the grafting yields were not calculated for these reactions. The *M*_n_ and the weight fraction of the shell material in the copolymers could be estimated from their known composition (determined by ^1^H NMR analysis) along with the known *M*_n_ value of the PBG substrate as described earlier [16], but in this case the signal from the PBG core is lost (due to its reduced mobility) in the ^1^H NMR analysis. Therefore, the grafting yields were determined from the area ratio of the peaks obtained with the DRI detector. In this approach the graft copolymer peak area is divided by the total peak area for the graft copolymer and the unreacted side chain peaks. The grafting yields thus obtained are unfortunately overestimated, since a fraction of the graft copolymer peak response is due to the PBG core, which has a higher d*n*/d*c* value than PEO (PBG d*n*/d*c* = 0.099 versus PEO d*n*/d*c* = 0.044 in DMF).

#### Dynamic Light Scattering Measurements

DLS measurements were performed on the chain end CuAAC-grafted arborescent copolymers in DMF and in PBS to characterize their solution properties. The first- and second-order hydrodynamic diameters obtained are provided in Table 6, and the corresponding CONTIN size distributions are supplied in the Supporting Information (Figures S1–S3). There is good agreement between the *d*_h1_ and *d*_h2_ values for all the samples in DMF, which is also reflected in comparatively narrow size distributions. Sample G3PBG-*eg*-PGly has *d*_h1_ and *d*_h2_ values similar to G2PBG-*eg*-PGly in DMF, as observed previously for a series of arborescent copolymers with a randomly grafted PGlyAc shell [16]. The smaller than expected *d*_h1_ and *d*_h2_ values for G3PBG-*eg*-PGly could be due to the lower weight fraction of polyglycidol in that sample (last column in Table 5), allowing the PGly chains to adopt a more coiled conformation to shield the PBG core from the surrounding environment. The arborescent copolymers containing PEO display decreasing *d*_h1_ and *d*_h2_ values for increasing generation numbers. This trend was also observed when comparing the SEC traces for these samples, where G1PBG-*eg*-PEO was eluted at a lower volume than both G2PBG-*eg*-PEO and G3PBG-*eg*-PEO. Despite this unusual trend, the differences between the *d*_h1_ and *d*_h2_ values for each sample remain small (≤4 nm), indicating that these structures have a uniform size in DMF.

The DLS results in PBS for the chain end CuAAC-grafted arborescent copolymer samples are more promising than for the random CuAAC-grafted samples in terms of unimolecular micelle formation. For example, G1PBG-*eg*-PGly and G2PBG-*eg*-PGly have much smaller *d*_h2_ values (54 and 53 nm, respectively) than their randomly-grafted counterparts (*d*_h2_ values of 199 and 67 nm for G1PBG-*g*-PGly and G2PBG-*g*-PGly, respectively). However sample G1PBG-*eg*-PGly has diameters larger than expected in PBS, and a large (24 nm) difference between *d*_h1_ and *d*_h2_, consistent with the presence of aggregated species. This was confirmed by CONTIN analysis (Figure S1), yielding a bimodal distribution corresponding to what are likely unimolecular micelles (with a population around 20 nm) and aggregated species (above 100 nm). While the size distribution for sample G1PBG-*eg*-PEO is unimodal in DMF and in PBS, the populations are rather large in comparison to the G1PBG substrate (ca. 10 nm) and centered around 40 nm and 60–70 nm (Figure S1), respectively, which is consistent with the formation of aggregates in PBS, and possibly also in DMF. As before, aggregate formation by the G1 copolymers is attributed to the relatively open structure of these molecules facilitating interactions between the G1PBG cores. Interestingly, samples G2PBG-*eg*-PEO, G3PBG-*eg*-PGly, and G3PBG-*eg*-PGly have comparable *d*_h1_ and *d*_h2_ values in PBS and in DMF, and displayed modest size increases when the hydrophilic shell components were grafted onto the PBG substrates (Figures S2 and S3), suggesting that these arborescent copolymers behave like unimolecular micelles in PBS. 

It is clear from the DLS data in PBS that chain-end CuAAC grafting is more useful for the synthesis of arborescent copolymers containing PEO: not only are all the copolymers soluble, but there is good agreement between the *d*_h1_ and *d*_h2_ values for each generation, and the CONTIN size distributions are unimodal although, as stated above, G1PBG-*eg*-PEO appears to be aggregated. These trends were also observed when comparing arborescent copolymers obtained by the random versus chain-end carbodiimide-promoted peptide coupling techniques [16]: the randomly-grafted arborescent copolymers containing PEO were either insoluble or displayed extensive aggregation, whereas the chain end-grafted copolymers yielded unimolecular micelles. Given the good agreement between the *d*_h1_ and *d*_h2_ values in the DLS results for G1PBG-*eg*-PEO and G2PBG-*eg*-PEO in both DMF and PBS, it seems likely that artefacts occurred in the SEC measurements of these copolymers (Figure 7). While the exact nature of these artefacts is not clear, they could be related to aggregation and/or column interactions during the SEC measurements.

## 4. Conclusions

The synthesis of well-defined arborescent copolymers consisting of linear chain segments grafted onto PBG cores by copper-catalyzed azide–alkyne Huisgen cycloaddition (CuAAC) chemistry was investigated. Well-defined linear polyglycidol, poly(ethylene oxide), and poly(2-trimethylsilylethyl acrylate) with terminal azide functionalities were synthesized by different polymerization techniques and chain end modification. Arborescent PBG with alkyne functionalities, either randomly distributed or at the chain ends, were derived from the corresponding carboxylic acid-functionalized substrates and propargylamine by carbodiimide activation.

Arborescent copolymers were synthesized by grafting either PGly, PEO, or P(HEA-TMS) chains onto the PBG cores through the CuAAC reaction. Successful CuAAC reactions were achieved with copper(II) sulfate and sodium ascorbate as catalyst, with grafting yields varying from 29% to 65%, depending on the PBG substrate and linear chain segments employed. Well-defined arborescent copolymers (*M*_w_/*M*_n_ ≤ 1.14) were obtained with the exception of two samples, G1PBG-*eg*-PEO and G2PBG-*eg*-PEO, characterized by *M*_w_/*M*_n_ values of 1.30 and 1.23, respectively.

The dynamic light scattering measurements revealed that even though the randomly grafted G1 and G2 arborescent copolymers with PHEA and PGly shells were dispersible in aqueous PBS solutions, they yielded either large aggregates or bimodal size distributions, while the analogous PEO copolymers were insoluble. Aggregation was arguably present even in the non-selective solvent DMF for most of the G1 copolymers, both randomly and end-grafted, presumably due to their open structure. In contrast, other copolymers with a more rigid PBG core (e.g., G2PBG-*eg*-PEO, G3PBG-*eg*-PGly, G3PBG-*eg*-PEO) were characterized by a modest size increase in DMF relatively to their parent PBG substrates, comparable dimensions in DMF and in PBS, and unimodal CONTIN size distributions. These samples, therefore, appear to behave like unimolecular micelles based on both their average hydrodynamic diameters and the CONTIN size distributions, or if aggregation is present, it is not significantly higher in aqueous PBS solutions than in DMF. Further confirmation for the unimolecular nature of the micelles could be obtained by comparing the results of static light scattering measurements in DMF and in PBS solutions for these copolymers. This will be the topic of a future investigation. Similar trends in the aggregation behavior were observed for analogous arborescent copolymers obtained by carbodiimide coupling, in particular when comparing the solubility of randomly vs. terminally grafted copolymer systems [16]. The lower aqueous dispersibility of the randomly-grafted and the lower generation copolymers was explained by less efficient shielding of van der Waals interactions between the cores, due to a more diffuse interface between the hydrophobic core and the hydrophilic shell components.

## Figures and Tables

**Table 1:** Characteristics of the linear polymers with terminal azide functionalities.

Polymer	SEC ^a^		^1^H NMR	
	*M*_n_ (g/mol)	*M*_w_/*M*_n_	*X*_n_^b^	*M*_n_ (g/mol) ^c^
PGlyAc	14,100	1.06	-	-
	*M*_n_^app^ (g/mol)	*M*_w_/*M*_n_^app^		
PGly	10,200	1.14	-	-
PEO	5900	1.08	11.3	5000
P(HEA-TMS)	9500	1.21	57.3	10,800

^a^ PGlyAc analyzed on the triple detector system using d*n*/d*c* = 0.045 mL/g in THF [19]; PEO and P(HEA-TMS) analyzed with a DRI detector in DMF, so only apparent values are reported; ^b^ number-average degree of polymerization; ^c^ *M*_n_ calculated from *X*_n_.

**Table 2:** Characteristics of randomly alkyne-functionalized G0PBG substrates CuAAC-grafted with various α-azide side chains using CuSO_4_/NaAsc.

Copolymer ^a^	PBG Substrate		*G*_y_^d^	Graft Copolymer			
	*M*_n_ (g/mol) ^b^	% Alkyne ^c^		*M*_n_ (g/mol) ^b^	*M*_w_/*M*_n_^b^	*f*_n_^e^	% Shell ^f^
G0PBG-*g*-P(HEA-TMS)	51,000	18	57	324,000	1.17	25	84
G0PBG-*g*-PGly	51,000	18	93	331,000	1.02	40	85
G0PBG-*g*-PEO	51,000	18	54	172,000	1.09	24	70

^a^ All reactions done with 1:1 azide to alkyne ratio; ^b^ absolute values from SEC-MALLS analysis in DMF; ^c^ functionalization level from ^1^H NMR analysis; ^d^ grafting yield: fraction of side chains attached to the substrate; ^e^ branching functionality: number of side chains added in the last grafting cycle; ^f^ weight fraction of shell material, calculated from the difference in absolute molecular weights of the copolymer and the substrate.

**Table 3:** Characteristics of arborescent G1 and G2 copolymers obtained by random CuAAC-grafting.

Copolymer ^a^	PBG Substrate		*G*_y_^d^	Graft Copolymer			
	*M*_n_ (g/mol) ^b^	% Alkyne ^c^		*M*_n_ (g/mol) ^b^	*M*_w_/*M*_n_^b^	*f*_n_^e^	% Shell ^f^
G1PBG-*g*-P(HEA-TMS)	322,000	19	27	1.2 × 10^6^	1.14	80	73
G2PBG-*g*-P(HEA-TMS)	1.1 × 10^6^	32		Reaction Failed			
G1PBG-*g*-PGly	322,000	19	20	734,000	1.09	58	56
G2PBG-*g*-PGly	1.1 × 10^6^	32	13	2.8 × 10^6^	1.03	234	60
G1PBG-*g*-PEO	322,000	19	57	1.2 × 10^6^	1.13	166	72
G2PBG-*g*-PEO	1.1 × 10^6^	32	8	1.8 × 10^6^	1.08	140	38

^a^ All reactions done with a 1:1 azide to alkyne ratio; ^b^ absolute values from SEC-MALLS in DMF; ^c^ functionalization level determined from ^1^H NMR analysis; ^d^ grafting yield: fraction of side chains attached to the substrate; ^e^ branching functionality: number of side chains added in the last grafting cycle; ^f^ weight fraction of shell material, from the difference in absolute molecular weights of the copolymer and the substrate.

**Table 4:** DLS measurements for randomly CuAAC-grafted arborescent copolymers.

Copolymer	PBG Core (DMF) ^a^		Graft Copolymer (DMF) ^a^		Graft Copolymer (PBS) ^b^	
	*d*_h1_ (nm) ^c^	*d*_h2_ (nm) ^d^	*d*_h1_ (nm) ^c^	*d*_h2_ (nm) ^d^	*d*_h1_ (nm) ^c^	*d*_h2_ (nm) ^d^
G1PBG-*g*-PHEA	11.7 ± 1	10.4 ± 1	61.8 ± 1	47.6 ± 1	128 ± 1	104 ± 1
G1PBG-*g*-PGly	11.7 ± 1	10.4 ± 1	78.5 ± 1	65.8 ± 1	221 ± 1	199 ± 1
G2PBG-*g*-PGly	18.7 ± 1	17.5 ± 1	39.0 ± 1	35.1 ± 1	94.9 ± 1	67.4 ± 1
G1PBG-*g*-PEO	11.7 ± 1	11.4 ± 1	24.2 ± 1	20.5 ± 1	insoluble	
G2PBG-*g*-PEO	18.7 ± 1	17.5 ± 1	30.4 ± 1	27.8 ± 1	insoluble	

^a^ DMF with 0.05% LiCl to prevent aggregation; ^b^ phosphate-buffered saline (pH 7.4); ^c^ hydrodynamic diameter from 1st order analysis of the correlation function; ^d^ diameter from second-order analysis.

**Table 5:** Characteristics of arborescent copolymers obtained by chain-end CuAAC grafting.

Copolymer ^a^	PBG Substrate		*G*_y_^d^	Graft Copolymer			
	*M*_n_ (g/mol) ^b^	% Alkyne ^c^		*M*_n_ (g/mol) ^b^	*M*_w_/*M*_n_^b^	*f*_n_^e^	% Shell ^f^
G1PBG-*eg*-PGly	322,000	7	98	906,000	1.09	89	69
G2PBG-*eg*-PGly	1.1 × 10^6^	12	47	3.2 × 10^6^	1.02	294	65
G3PBG-*eg*-PGly	3.0 × 10^6^	11	30	6.3 × 10^6^	1.01	467	52
G1PBG-*eg*-PEO	280,000	7	50 ^g^	^h^	1.30		
G2PBG-*eg*-PEO	1.1 × 10^6^	12	36 ^g^	^h^	1.23		
G3PBG-*eg*-PEO	3.0 × 10^6^	11	24	4.8 × 10^6^	1.04	738	38

^a^ All reactions done with a 1:1 azide to alkyne ratio; ^b^ absolute values from SEC-MALLS in DMF; ^c^ functionalization level from ^1^H NMR analysis; ^d^ grafting yield: fraction of side chains attached to the substrate; ^e^ branching functionality: number of side chains added in the last grafting cycle; ^f^ weight fraction of shell material, from the difference in absolute molecular weight of copolymer and substrate; ^g^ grafting yield from the peak area ratio in the DRI response; ^h^ absolute molecular weight not determined.

**Table 6:** DLS measurements for chain end CuAAC-grafted arborescent copolymers.

Copolymer	PBG Core (DMF) ^a^		Graft Copolymer (DMF) ^a^		Graft Copolymer (PBS) ^b^	
	*d*_h1_ (nm) ^c^	*d*_h2_ (nm) ^d^	*d*_h1_ (nm) ^c^	*d*_h2_ (nm) ^d^	*d*_h1_ (nm) ^c^	*d*_h2_ (nm) ^d^
G1PBG-*eg*-PGly	11.7 ± 1	10.0 ± 1	30.3 ± 1	26.3 ± 1	78.9 ± 1	54.6 ± 1
G2PBG-*eg*-PGly	18.9 ± 1	17.3 ± 1	39.7 ± 1	35.5 ± 1	72.5 ± 1	53.0 ± 1
G3PBG-*eg*-PGly	28.4 ± 1	26.8 ± 1	39.9 ± 1	38.9 ± 1	43.5 ± 1	39.8 ± 1
G1PBG-*eg*-PEO	11.7 ± 1	10.0 ± 1	46.8 ± 1	42.1 ± 1	61.7 ± 1	54.4 ± 1
G2PBG-*eg*-PEO	18.9 ± 1	17.3 ± 1	43.9 ± 1	39.4 ± 1	45.8 ± 1	41.7 ± 1
G3PBG-*eg*-PEO	28.4 ± 1	26.8 ± 1	38.3 ± 1	35.4 ± 1	56.9 ± 1	50.5 ± 1

^a^ DMF with 0.05% LiCl to prevent aggregation; ^b^ phosphate-buffered saline (pH 7.4); ^c^ hydrodynamic diameter from 1st order analysis of the correlation function; ^d^ diameter from 2nd order analysis.

## Supplementary Materials

The following are available online at www.mdpi.com/2073-4360/9/10/540/s1.
