# Supplementary material for: Arborescent Unimolecular Micelles: Poly(γ-Benzyl l-Glutamate) Core Grafted with a Hydrophilic Shell by Copper(I)-Catalyzed Azide–Alkyne Cycloaddition Coupling
Source: Polymers (Basel). 2017 Oct 23;9(10):540. doi: 10.3390/polym9100540 (PMC6418952; doi:10.3390/polym9100540)
Supplement: Supplementary file 1 [file polymers-09-00540-s001.pdf]

Arborescent Unimolecular Micelles: Poly(γ-Benzyl L-Glutamate) Core Grafted with a Hydrophilic Shell by Copper(I)-Catalyzed Azide–Alkyne Cycloaddition Coupling

Mario Gauthier,* Greg Whitton

Supporting Information

Experimental Procedures

Synthesis of α-Azido PGlyAc

2,3-Epoxy-1-(1-ethoxyethoxy)propane (glycidol acetal) was synthesized as described by Fitton et al. [17] and was stored under N_2 at 4 °C. The procedure used to obtain an α-azido poly(glycidol acetal) sample (target M_n = 10,000 g/mol) was adapted from Gervais et al. [18]. The initiator, tetrabutylammonium azide (0.57 g, 2.00 mmol; Sigma-Aldrich, Oakville, ON, Canada), was dried by three cycles of azeotropic distillation with dry toluene under vacuum, redissolved in 30 mL of toluene and stored under N_2 in a glass ampoule sealed with a polytetrafluoroethylene (PTFE) stopcock. A 1-L, 5-neck round-bottomed flask (RBF) was evacuated under high-vacuum, flame-dried, and purged with N_2. Dry toluene (240 mL) was added before cooling to -30 °C with dry ice in a 2-propanol/water bath. Glycidol acetal (20.0 g, 0.135 mol, target X_n = 68.5, freshly distilled over triisobutylaluminum), the initiator solution, and triisobutylaluminum (2.0 mL of solution, 2.0 mmol; 1.0 M in hexanes, Sigma-Aldrich, Oakville, ON, Canada) were then added in succession, and the cooling bath was removed to allow the reaction to proceed at room temperature (RT) overnight. Degassed ethanol was added to terminate the reaction. The toluene was removed, the polymer was redissolved in 100 mL of diethyl ether (≥99.7%, Sigma-Aldrich, Oakville, ON, Canada) and left in the refrigerator overnight so that the salts formed in the reaction would precipitate. After filtration with a 0.45 μm PTFE filter, the diethyl ether was removed under vacuum to achieve a constant sample weight. Yield: 17.9 g (90%). IR: sharp N_3 stretch at 2102 cm^{-1}. SEC (THF): M_n = 14,100 g/mol, M_w/M_n= 1.06; ^{1}H NMR (300 MHz, $CDCl_3$) δ: 4.66 (q, 1H), 3.67–3.35 (m, 7H), 1.24 (d, 3H), 1.15 (t, 3H).

Synthesis of α-Azido PGly

The procedure for the removal of the acetal protecting group from α-azide poly(glycidol acetal) was adapted from Mendrek et al. [19]. α-Azide PGlyAc (7.0 g, 0.048 mol acetal, 1 eq.) was dissolved in 350 mL of *N*,*N*-dimethylformamide (DMF) and placed in a 1-L RBF with a stirring bar. A concentrated HCl solution (20 mL, 11.6 M, 0.24 mol HCl, 5 eq.) was then added with rapid stirring. After 30 min at RT, enough saturated $NaHCO_3$ solution (ca. 40 mL) was added to neutralize the HCl. The DMF and water were removed under vacuum, the polymer was redissolved in 30 mL of ethanol and salts were removed by filtration. The solution was purified by dialysis in a 1000 g/mol molecular weight cut-off (MWCO) bag against ethanol (500 mL) overnight, and then against methanol for 2 h. The solution was finally evaporated and the polymer was dried in a vacuum oven at 50 °C overnight. Yield: 2.6 g (74%). SEC (DMF): M_n^{app} = 10,200 g/mol, M_w/M_n^{app} = 1.14 (DRI), IR: sharp N_3 stretch at 2102 cm^{-1}. ^{1}H NMR (300 MHz, d_6-DMSO) δ: 4.66 (q, 1H), 3.67–3.35 (m, 5H).

Synthesis of ω-Tosyl PEO

Linear PEO monomethyl ether with M_n = 5000 g/mol (Polysciences, Warrington, PA, USA; X_n = 113,

10.0 g, 0.002 mol –OH, 1 eq.) was added to 100 mL of dichloromethane (DCM) in a 250-mL RBF. *p*-Toluenesulfonyl chloride (7.63 g, 0.04 mol, 20 eq.; Reagent Grade, Sigma-Aldrich, Etobicoke, ON, Canada) and pyridine (3.2 mL, 0.04 mol, 20 eq.; ACS Reagent, Sigma-Aldrich, Etobicoke, ON, Canada) were dissolved in 40 mL of DCM in a 250-mL RBF with stirring. The ω-hydroxy PEO solution was added to the *p*-toluenesulfonyl chloride solution and stirred overnight at RT under N_2. The polymer was precipitated in cold diethyl ether, recovered by filtration, and purified by dissolution in methanol, precipitation in ether and drying under vacuum overnight. Since according to ^{1}H NMR analysis only 85% conversion was achieved, the tosylation reaction was repeated to attain full conversion. Yield: 9.6 g (96%). ^{1}H NMR (300 MHz, $CDCl_3$) δ: 9.29 (d, 1H), 8.88 (q, 4H), 8.45 (m, 3H), 7.99–7.94 (m, 6H), 7.80 (d, 4H), 7.32 (d, 2H), 7.25–7.15 (d, 4H), 4.15–4.10 (t, 2H), 3.85–3.35 (b, 444H), 2.43 (s, 1H), 2.34 (s, 2H).

Synthesis of ω-Azido PEO

ω-Tosyl PEO (9.6 g, 1.92 mmol ω-tosyl, 1 eq.) was dissolved with stirring in 50 mL of DMF in a 100-mL RBF and sodium azide (NaN_3, 2.5 g, 38.4 mmol, 20 eq.; ReagentPlus, Sigma-Aldrich, Etobicoke, ON, Canada) was added, the RBF was purged with N_2 and the reaction was stirred at RT for 48 h. Deionized water was then added until the solution became clear, and stirring was continued for 30 min in a well-ventilated fume hood to allow hydrazoic acid vapors potentially present to escape. The polymer was recovered as described for ω-tosyl PEO. Yield: 7.5 g (78%). IR: azide stretch at 2115 cm^{-1}. ^{1}H NMR (300 MHz, $CDCl_3$) δ: 3.85–3.35 (b, 4H).

Synthesis of 2-(Trimethylsilyloxy)ethyl Acrylate (HEA-TMS)

To achieve the controlled polymerization of 2-hydroxyethyl acrylate (HEA) via ATRP, the hydroxyl group was protected as a trimethylsilyl ether by a procedure adapted from Mühlebach et al. [20]. Freshly distilled HEA (50 mL, 0.435 mol, 1 eq.; 96%, Sigma-Aldrich, Etobicoke, ON, Canada), dichloromethane (500 mL), and triethylamine (73 mL; ≥99%, Sigma-Aldrich, Etobicoke, ON, Canada) were stirred in a 1-L RBF under N_2 at 0 °C and trimethylsilyl chloride (61 mL, 0.479 mol, 1.1 eq.; ≥99%, Sigma-Aldrich, Etobicoke, ON, Canada) was added over 20 min. After allowing the reaction to warm to RT, the salts formed were removed by filtration. Dichloromethane was evaporated and the solution was filtered again, diluted with ethyl acetate (300 mL), and washed 3 times with 300 mL of deionized water. The organic phase was dried over $MgSO_4$, the solvent was removed, and the product was distilled under vacuum to give a colorless liquid. Yield: 61.2 g (74%). ^{1}H NMR (300 MHz, $CDCl_3$) δ: 6.43–6.37 (d, 1H), 6.17–6.07 (q, 1H), 5.82–5.79 (d, 1H), 4.22–4.19 (t, 2H), 3.81–3.78 (t, 2H), 0.10 (s, 9H).

Synthesis of ω-Bromo P(HEA-TMS)

The polymerization of HEA-TMS was conducted in a pre-dried Schlenk flask with a magnetic stirring bar. Copper(I) bromide (CuBr, 0.155 g, 1.08 mmol, 1 eq.; 99.999%, Sigma-Aldrich, Etobicoke, ON, Canada) was loaded in the flask under N_2. *N,N,N′,N′′,N′′*-Pentamethyldiethylenetriamine (PMDETA, 0.450 mL, 2.16 mmol, 2 eq.; 99%, Sigma-Aldrich, Etobicoke, ON, Canada) was added, followed by HEA-TMS (16.2 g, 86.2 mmol, 80 eq.) and methyl 2-bromopropionate (0.120 mL, 1.08 mmol, 1 eq.; 98%, Sigma-Aldrich, Etobicoke, ON, Canada). The target degree of polymerization (X_n) was 50, corresponding to M_n = 9400 g/mol at 63% monomer conversion. Three freeze-pump-thaw (FPT) cycles were performed, the flask was purged with N_2 and placed in an oil bath at 90 °C with stirring. To monitor monomer conversion, a sample was removed with a syringe after 20 min and cooled in liquid N_2 for ^{1}H NMR analysis. The polymerization was stopped after 35 min by opening the Schlenk flask to the air and cooling in liquid N_2. Water was added to precipitate the polymer which was isolated by centrifugation, redissolved in 50 mL of ethyl acetate, and dried over $MgSO_4$. The solvent was then removed under

vacuum overnight. The polymer was stored in a refrigerator (4 °C) after dissolution in diethyl ether (100 mL). Yield: 9.2 g (78%), SEC (DMF): M_n^{app} = 9500 g/mol, M_w/M_n^{app} = 1.21 (DRI), ^{1}H NMR (300 MHz, $CDCl_3$): X_n = 57.3, M_n = 10,800 g/mol, δ: 6.45–6.39 (m, 4H), 6.19–6.09 (m, 4H), 5.84–5.80 (m, 4H), 4.07 (b, 115H), 3.73 (b, 115H), 2.33 (b, 57H), 1.90–1.40 (b, 115H), 1.13 (b, 3H), 0.10 (s, 516H).

Synthesis of ω-Azido P(HEA-TMS)

ω-Bromo P(HEA-TMS) (5.0 g, 0.463 mmol, 1 eq.) was loaded in a dry 100-mL N_2-purged RBF with a stirring bar. DMF (50 mL) was added, followed by sodium azide (NaN_3, 0.60 g, 9.26 mmol, 20 eq.). The reaction was stirred overnight at RT. To minimize cleavage of the TMS group by the hydrazoic acid produced when sodium azide is exposed to water, the DMF solution was decanted to remove insoluble NaN_3, poured into 200 mL of water, and centrifuged. The polymer was then redissolved in 50 mL of ethyl acetate, dried over $MgSO_4$, the ethyl acetate was evaporated and the polymer was dried under vacuum overnight. The product was stored in a refrigerator (4 °C) as a solution in diethyl ether. Yield: 3.5 g (70%). IR: azide stretch at 2117 cm^{-1}. ^{1}H NMR (300 MHz, $CDCl_3$) δ: 6.45–6.39 (m, 4H), 6.19–6.09 (m, 4H), 5.84–5.80 (m, 4H), 4.07 (b, 115H), 3.73 (b, 115H), 2.33 (b, 57H), 1.90–1.40 (b, 115H), 1.13 (b, 3H), 0.10 (s, 464H).

Results and Discussion

Synthesis of α-Azido Polyglycidol

The actual M_n determined by size exclusion chromatography was 14,100 g/mol, higher than the target M_n = 10,000 g/mol. This is attributed to tetrabutylammonium azide losses during azeotropic drying of the initiator prior to the polymerization; the molecular weight distribution of the polymer was nevertheless narrow (M_w/M_n = 1.06). Due to overlapping signals in the spectrum, an accurate estimate of the degree of polymerization could not be obtained by ^{1}H NMR analysis. One advantage of CuAAC coupling is that many functional groups, other than azides or alkynes, do not interfere with the reaction. This allowed removal of the acetal protecting groups prior to the coupling reaction. The corresponding α-azido polyglycidol sample (α-azido PGly, M_n = 7100 g/mol) was characterized by IR spectroscopy to verify that the azide functionality was still present. The IR spectrum obtained for PGlyAc had a sharp azide stretch near 2100 cm^{-1}, that remained strong in the α-azido PGly spectrum, in addition to a broad peak between 3500–3000 cm^{-1} for the free hydroxyl groups in α-azido PGly.

Synthesis of ω-Azido Poly(ethylene oxide)

A commercial poly(ethylene oxide) (PEO) monomethyl ether sample with M_n = 5000 g/mol, with one ω-hydroxyl group, was converted to ω-azido PEO via an ω-tosyl PEO intermediate. In addition to anhydrous conditions, a large excess of *p*-toluenesulfonyl chloride (20 equivalents) and an extended reaction time were used to achieve a high conversion. Unfortunately even under these conditions only 85% conversion was achieved, so a second reaction step was necessary for full conversion. The ^{1}H NMR spectrum obtained for ω-tosyl PEO had a peak at 4.15 ppm for to the -CH_2- protons adjacent to the ω-tosyl functional group. The conversion level calculated by integration of the signal at 4.15 ppm and the peak for the backbone protons at 3.6 ppm yielded a ratio of 1:113, corresponding to 100% conversion of ω-hydroxyl PEO to ω-tosyl PEO. Sodium azide was then used to convert the tosylated polymer to ω-azido PEO. The reaction was allowed to proceed for 48 h to ensure complete conversion. The signal in the ^{1}H NMR spectrum for the protons adjacent to the tosyl group (4.15 ppm) disappeared after the reaction, confirming that the group had been displaced. No signals were visible for the protons adjacent to the azido group, due to overlapping signals from the protons in the polymer backbone near 3.6 ppm.

The presence of the azide functionality on the PEO chains was confirmed by IR analysis, through an azide stretch clearly visible at 2116 cm^{-1}.

Synthesis of ω-Azido Poly(2-trimethylsilylethyl acrylate)

While the ATRP of 2-hydroxyethyl acrylate (HEA) in its unprotected form has been reported, it was proposed that better control over the polymerization reaction and higher conversions could be achieved if the hydroxyl group were protected [20]. The HEA monomer was therefore protected with a labile trimethylsilyl (TMS) group in this work. The polymerization of HEA-TMS was monitored by ^1H NMR analysis as the monomer conversion, calculated from the peak integration ratio for the alkene protons of the monomer (6.5–5.7 ppm) and the trimethylsilyl protons present on both the monomer and the polymer at 0.1 ppm. After 20 min, 49% monomer conversion had been reached. Based on a monomer to initiator ratio (M/I) of 80, 49% monomer conversion should correspond to a degree of polymerization $X_n = 39$. The ratio of the polymer backbone $-CH_2-$ protons (2.0–1.3 ppm) to the methyl initiator protons (1.1 ppm) provided an X_n value of 35. The polymerization was stopped after 35 min based on the results from the t = 20 min sample. At t = 35 min the monomer conversion (74%) corresponded to $X_n = 59$, whereas NMR analysis yielded $X_n = 57$. After sample purification $X_n = 57.3$ was obtained, corresponding to $M_n = 10{,}800$ g/mol.

The conversion of ω-bromo P(HEA-TMS) to ω-azido P(HEA-TMS) was performed with NaN_3 in DMF over 48 h. The presence of the azide functionality could not be confirmed by ^1H NMR analysis due to overlapping peaks from the polymer that interfered with the protons adjacent to the ω-bromo and ω-azido functionalities. IR analysis nevertheless confirmed the presence of an azide group on the polymer with a small peak for the azide stretch at 2117 cm^{-1}. The ^1H NMR spectrum for ω-azido P(HEA-TMS) revealed that approximately 7% of the TMS protecting groups were cleaved during the azidation reaction. This is attributed to the presence of hydrazoic acid, produced by trace amounts of water in the DMF used for the reaction. ω-Azido P(HEA-TMS) was stored in diethyl ether until it was used in the coupling reaction, to avoid potential cross-linking of the unprotected 2-hydroxyethyl acrylate repeating units.

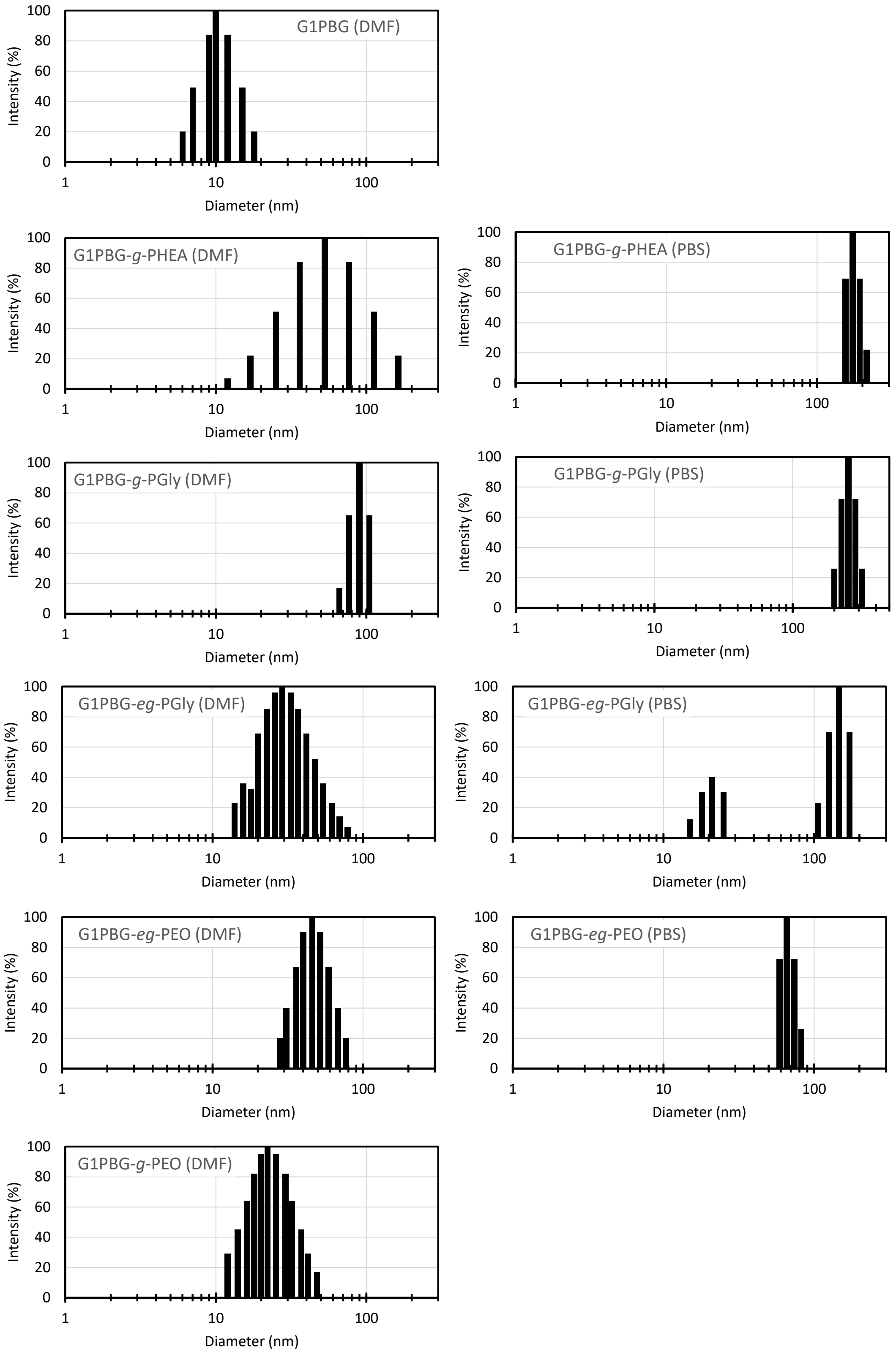

Figure S1: CONTIN size distributions – G1PBG and copolymers.

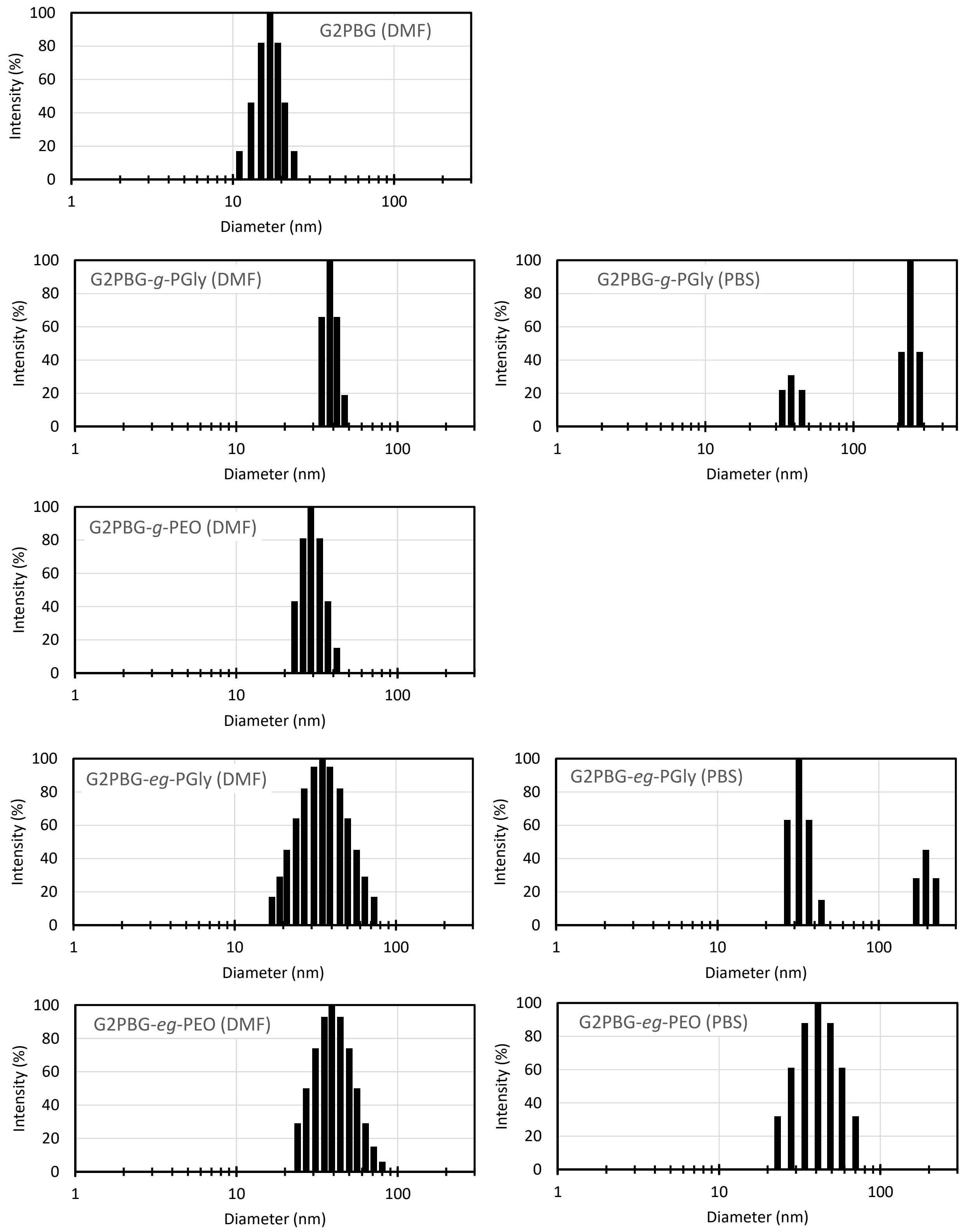

Figure S2: CONTIN size distributions – G2PBG and copolymers.

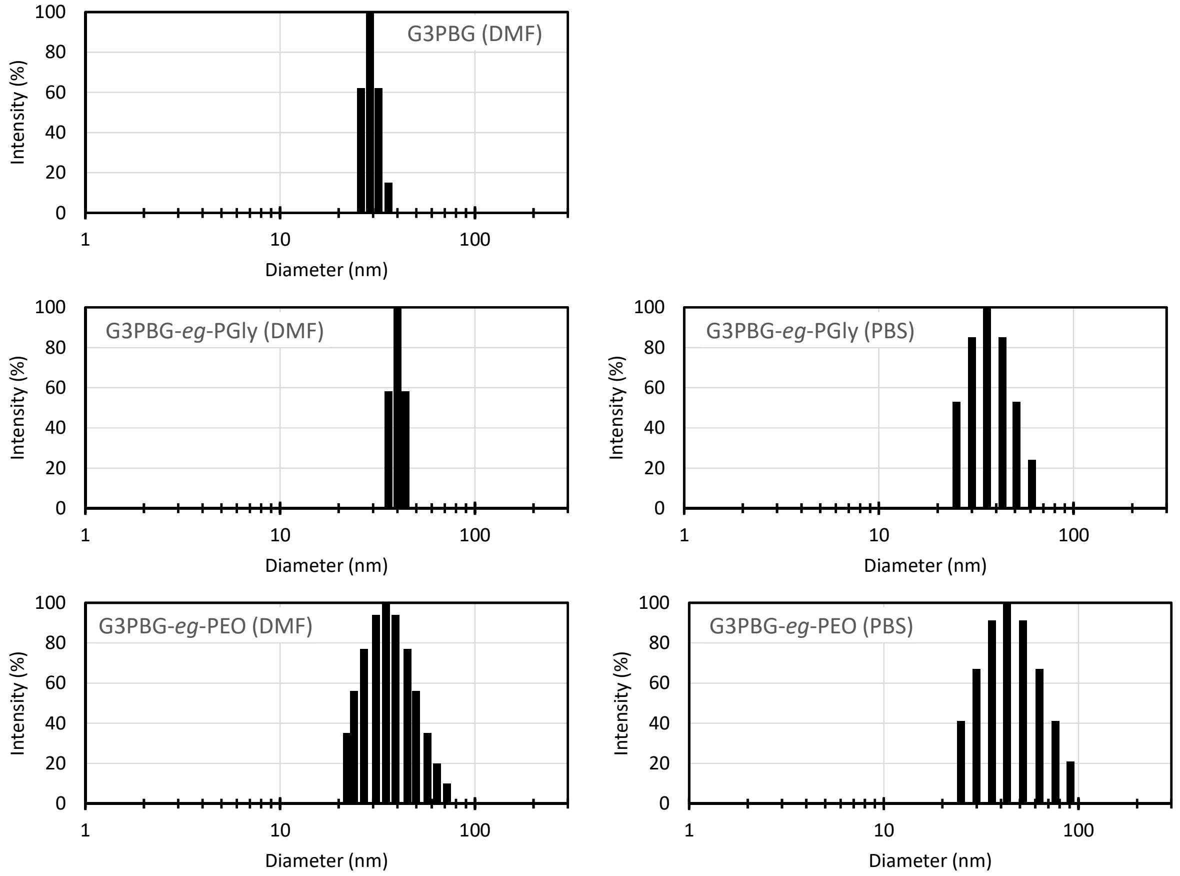

Figure S3: CONTIN size distributions – G3PBG and copolymers.
